# Predictive value of lactate/albumin ratio for death and multiple organ dysfunction syndrome in patients with sepsis

**DOI:** 10.5937/jomb0-46947

**Published:** 2024-06-15

**Authors:** Qiuqiang Chen, Haichao Zhan, Junyu Chen, Junde Mo, Shuwei Huang

**Affiliations:** 1 Central People's Hospital of Zhanjiang, Department of Second Ward of Intensive Care Medicine, Zhanjiang, Guangdong Province, China; 2 Central People's Hospital of Zhanjiang, Department of First Ward of Intensive Care Medicine, Zhanjiang, Guangdong Province, China; 3 Central People's Hospital of Zhanjian, Department of Emergency, Zhanjiang, Guangdong Province, China

**Keywords:** sepsis, multiple organ dysfunction syndrome, mortality, lactate, low albumin, sepsa, višestruki sindrom organske disfunkcije, mortalitet, laktat, niski albumin

## Abstract

**Background:**

Multiple organ dysfunction syndrome (MODS) is common after sepsis and increases mortality. Lactate (Lac) can assess the prognosis of patients. Albumin (Alb) is closely associated with inflammatory response in sepsis patients. This work evaluated the predictive value of Lac/Alb for prognosis of sepsis patients.

**Methods:**

Data of 160 sepsis patients were retrospectively collected. Lac and Alb levels were measured upon admission, at 24 hours and 48 hours later. Using 0.45 as the cutoff value for Lac/Alb, patients were rolled into high-level (HL) and low-level (LL) groups. MODS rates and mortality rates were analyzed. Receiver operating characteristic (ROC) curves were utilized to evaluate the predictive value of 48-hour Lac/Alb for patient prognosis. Correlation between Lac/Alb and APACHE II and SOFA scores was assessed.

**Results:**

The 12-month follow-up revealed 52 deaths (32.5%), and MODS occurred in 49 cases (30.6%) on the 7th day. The MODS group possessed elevated Lac and Lac/Alb and decreased Alb to the N-MODS group (P<0.05), and similar results were observed by comparison the survival and death group (P<0.05). The sensitivity, specificity, and area under the ROC curve (AUC) of Lac/Alb in predicting MODS were 81.63%, 85.59%, and 0.89, respectively, while those in predicting death were 94.23%, 88.89%, and 0.91, respectively. Lac/Alb was positively correlated with APACHE II and SOFA scores (r=0.718 and 0.808, respectively).

**Conclusions:**

Lac/Alb was linked to MODS and mortality in sepsis patients and can be based to predict adverse outcomes.

## Abstract (sr)

**Uvod:**

Sindrom višeorganske disfunkcije (MODS) je čest nakon sepse i povećava mortalitet. Laktat (Lac) može proceniti prognozu pacijenata. Albumin (Alb) je usko povezan sa inflamatornim odgovorom kod pacijenata sa sepsom. Ovaj rad je procenio prediktivnu vrednost Lac/Alb za prognozu pacijenata sa sepsom.

**Metode:**

Retrospektivno su prikupljeni podaci od 160 pacijenata sa sepsom. Nivoi Lac i Alb su mereni po prijemu, 24 sata i 48 sati kasnije. Koristeći 0,45 kao graničnu vrednost za Lac/Alb, pacijenti su raspoređeni u grupe visokog (HL) i niskog nivoa (LL). Analizirane su stope MODS-a i stope mortaliteta. Krive operativnih karakteristika prijemnika (ROC) su korišćene za procenu prediktivne vrednosti 48-časovnog Lac/Alb za prognozu pacijenta. Procenjena je korelacija između Lac/Alb i APACHE II i SOFA rezultata.

**Rezultati:**

12-mesečno praćenje otkrilo je 52 smrti (32,5%), a MODS se dogodio u 49 slučajeva (30,6%) 7. dana. Grupa MODS je imala povišene Lac i Lac/Alb i smanjene vrednosti Alb u odnosu na N-MODS grupu (P<0,05), a slični rezultati su uočeni u poređenju sa grupom preživljavanja i smrti (P<0,05). Osetljivost, specifičnost i površina ispod ROC krive (AUC) Lac/Alb u predviđanju MODS-a bile su 81,63%, 85,59% i 0,89, respektivno, dok su one u predviđanju smrti bile 94,23%, 88,89% i 0,91, respektivno. Lac/Alb je bio u pozitivnoj korelaciji sa APACHE II i SOFA skorom (r=0,718 i 0,808, respektivno).

**Zaključak:**

Lac/Alb je povezan sa MODS-om i mortalitetom kod pacijenata sa sepsom i može se zasnivati na predviđanju neželjenih ishoda.

## Introduction

Sepsis features with a systemic inflammatory response, which is primarily triggered by infections [1]. It leads to a pathological and physiological imbalance in patients, and the systemic inflammatory response can, to a certain extent, cause damage to circulatory tissues or organs, potentially leading to organ failure [2] [3]. When a patient's body overreacts to an infection, the immune system releases a large amount of cytokines and chemical substances, causing the inflammation to spread throughout the body, resulting in the failure of multiple organ systems and the development of multiple organ dysfunction syndrome (MODS) [4]. MODS can potentially affect variousorgans, including the lungs, kidneys, heart, liver, digestive system, central nervous system, and more. Serum lactate (Lac) is a metabolic product of the body, primarily produced by red blood cells, skeletal muscles, and brain tissue. Elevated levels of Lac in the body can lead to lactic acidosis [5]. Lac is an important indicator for assessing the prognosis of patients suffering from severe infections and septic shock [6]. Serum albumin (Alb), synthesized by the liver, is abundant in the blood plasma. It exerts a crucial effect in physiological functions such as binding and transport, as well as maintaining blood colloid osmotic pressure [7]. Alb levels are closely related to inflammatory response in sepsis patients [8]. In critically ill sepsis patients, both Lac and Alb are important factors for evaluating prognosis. However, involvement of Lac/Alb ratio in predicting the prognosis of sepsis patients requires further investigation. Therefore, this study aimed to yield reference data for the predictive value of the Lac/Alb ratio in assessing the adverse prognosis of critically ill sepsis patients [9] [10].

## Materials and methods

### Study populations

A retrospective analysis was implemented on 160 sepsis patients the intensive care unit (ICU) of ** Hospital from January 2021 to June 2023, including 95 males and 65 females. They were 25–70 years old (55.1 ± 2.8 years old in average). Their duration of illness (DOI) ranged from 2 to 12 days, which was averaged as (7.3 ± 0.9) days. Populations enrolled had to satisfy all the following conditions: (1) meeting the symptoms of sepsis stipulated in the *Chinese Sepsis and Septic Shock Emergency Treatment Guidelines (2018)* [11]; (2) age 18 years; and (3) normal cognitive function. Patients with any of following conditions had to be excluded: (1) concurrent human immunodeficiency virus infection; (2) chronic diseases affecting vital heart, liver, or kidneys; (3) conditions like bone marrow suppression and leukopenia; (4) malignant tumors; (5) pregnancy or lactation; (6) history of urinary tract infections, biliary tract infections, gastrointestinal infections, etc.; and (7) external trauma [12] [13]. This study obtained approval from the ** Hospital Ethics Committee.

### Research methods

All patients had 2 mL of arterial blood collected at admission (0 h), 24 hours after admission, and 48 hours after admission. Low Alb and arterial Lac levels were determined using a Roche fully automated biochemical analyzer and a fully automated blood gas analyzer, respectively. Based on the measurements, the Lac/Alb ratio was calculated. Patients received bundled and standardized treatment following the sepsis guidelines, with a target mean arterial pressure (MAP) of 65 mm Hg, central venous pressure (CVP) maintained at 8–12 mm Hg, and a urinary output goal of 0.5 mL/kg/h for resuscitation. This work primarily focused on the probability of patients developing MODS (within 7 days, defined by severe sepsis with dysfunction in two or more organs) and mortality (within 12 months). Patients were categorized into MODS and non-MODS groups based on the occurrence of MODS and into survival and death groups based on mortality. Patient data recorded at admission included body mass index (BMI), body temperature, heart rate, medical history, mechanical ventilation, use of vasoactive drugs, complete blood count, blood biochemistry, electrolytes, MAP, Acute Physiology and Chronic Health Scoring System II (APACHE II) scores [9], and Sequential Organ Failure Assessment (SOFA) scores [10]. Oxygenation index (PaO_2_/FiO_2_) and central venous oxygen saturation (ScvO_2_) were also measured.

### Methods for statistical analysis

Data analysis was conducted using SPSS 19.0. Continuous variables were given as mean ± standard deviation. Between-group comparisons were performed using t-tests, and repeated measures analysis of variance was utilized within each group. Categorical data were presented as percentages, and between-group comparisons were conducted using the χ^2^ test. Survival curves for patients in HL and LL groups were Kaplan-Meier curves and were compared using the Log-Rank test. Furthermore, the receiver operating characteristic (ROC) curves were plotted to evaluate the predictive value of 48-hour Lac/Alb for progression of MODS or mortality in sepsis patients. The area under the ROC curve (AUC) was compared using the Z-test. Additionally, sensitivity, specificity, positive predictive value (PPV), and negative predictive value (NPV) were calculated. Further more, the Spearman rank correlation test was utilized to analyze correlation between Lac/Alb and APACHE II scores and SOFA scores. Statistical significance was defined as *P*<0.05.

## Results

### Basic characteristics of sepsis patients

Over the 12-month follow-up period, there were 52 deaths among sepsis patients (32.5%). MODS was observed in 13 cases (8.1%) on the first day and in 49 cases (30.6%) on the seventh day. At the 48-hour mark, the median values for serum Lac, Alb, and Lac/Alb were 6.82 mmol/L, 17.58 g/L, and 0.45, respectively. Using 0.45 as the cutoff value for Lac/Alb, this work analyzed the differences in baseline characteristics between patients in HL and LL groups (Table 1). The table displayed no visible differences (*P*>0.05) in age, BMI, body temperature, heart rate (HR), white blood cell count (WBC), red blood cell count (RBC), MAP, RBC transfusion rate, or vasoactive drug rate (VDR) between patients in HL and LL groups. Patients in HL group exhibited higher APACHE II scores, SOFA scores, mechanical ventilation rates (MVR), D-dimer (D-D) levels, and creatinine (Cre) levels, while their central venous pressure (CVP), PaO_2_/FiO_2_ ratio, S_cv_O_2_, and estimated glomerular filtration rate (eGFR) levels were lower, showing obvious differences with those in LL group (*P*<0.05).

**Table 1:** Basic characteristics of sepsis patients.

Item	LL group	HL group	*P*
Cases	107	53	
Age (years old)	60.9 ± 3.9	61.1 ± 4.3	0.768
BMI(kg/m^2^)	24.5 ± 1.8	24.6 ± 2.1	0.754
Body temperature (°C)	37.3 ± 0.6	37.1 ± 0.5	0.587
HR (times/min)	88.7 ± 1.5	90.6 ± 2.2	0.057
WBC (10^9^/L)	13.6 ± 2.3	15.1 ± 4.4	0.056
RBC (10^9^/L)	145.6 ± 10.1	150.7 ± 11.6	0.068
APACHE IIscore	21.5 ± 2.9	26.3 ± 3.0	<0.01
SOFAscore	7.3 ± 1.5	10.8 ± 0.7	<0.01
MAP (mm Hg)	71.8 ± 5.4	70.9 ± 4.8	0.305
CVP (mm Hg)	6.6 ± 1.2	5.3 ± 0.6	<0.01
PaO_2_/FiO_2_	181.6 ± 5.2	150.9 ± 4.3	<0.01
ScvO_2_ (%)	50.6 ± 2.3	45.1 ± 3.9	<0.01
MVR (%)	33 (30.8)	28 (52.8)	<0.01
RBC transfusion rate (%)	5 (4.7)	3 (5.7)	0.786
VDR (%)	8 (7.5)	5 (9.4)	0.679
Prothrombin time (PT) (s)	15.8 ± 2.0	17.9 ± 1.5	<0.01
D-D (ng/mL)	1836.9 ± 56.7	3351.4 ± 60.6	<0.01
Cre (μmol/L)	133.2 ± 18.6	210.9 ± 15.4	<0.01
eGFR (mL/min)	60.6 ± 9.8	44.3 ± 8.1	<0.01

### Changes in Lac/Alb levels of sepsis patients

**Figure 1:**
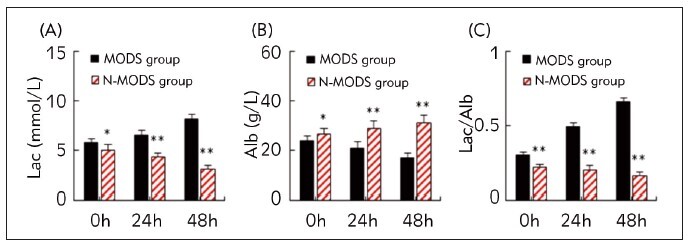
Changes in Lac/Alb for sepsis patients with and without MODS (A: Lac; B: Alb; C: Lac/Alb; * and ** suggested a great significance with P<0.05 and P<0.01 to the MODS group, respectively).

Differences in Lac, Alb, and Lac/Alb levels between sepsis patients with and without MODS were compared at admission, 24 hours after admission, and 48 hours after admission (Figure 1). In contrast to the condition at admission, Lac and Lac/Alb levels gradually increased, while Alb levels gradually decreased at 24 and 48 h in the MODS group. In contrast, in the N-MODS group, Lac and Lac/Alb experienced lowered levels and Alb exhibited an elevated level at these time points. When comparing the N-MODS group to the MODS group, it was observed that at 24 and 48 h, the Lac and Lac/Alb levels were greatly lower, while the Alb levels were elevated in N-MODS group (*P*<0.05).

**Figure 2:**
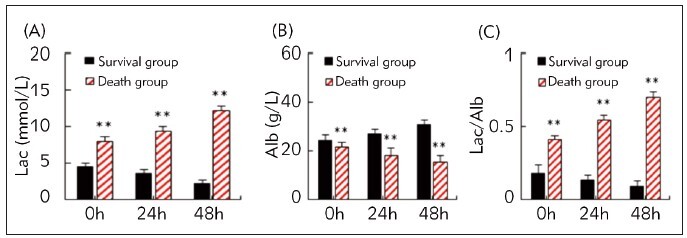
Changes in Lac/Alb for sepsis patients survived and died (A: Lac; B: Alb; C: Lac/Alb; ** suggested a great significance with P<0.01 to the survival group).

Figure 2 compared and analyzed the changes in Lac, Alb, and Lac/Alb levels between sepsis patients who survived and died. In the death group, serum Lac and Lac/Alb exhibited a slow upshifting trend, while Alb demonstrated an opposite trend at 0 h, 24 h, and 48 h in comparison to the conditions at admission. Conversely, in the survival group, Lac and Lac/Alb decreased, while Alb levels increased at these time points. The comparison disclosed that at all time points, Lac and Lac/Alb showed higher levels, while Alb levels were downshifted in the death group, exhibiting remarkable differences (*P*<0.05).

### Predictive value of Lac/Alb for prognosis of sepsis patients

**Figure 3:**
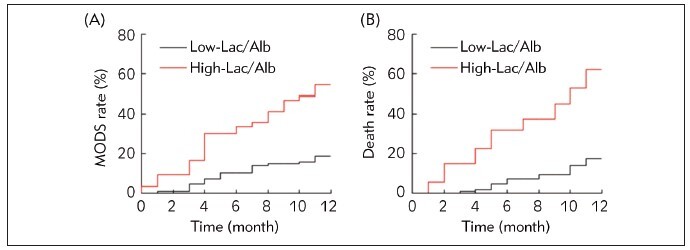
The survival curves for sepsis patients with different prognose (A: incidence of MODS; B: mortality).

The survival curves for sepsis patients with different prognoses were analyzed using the Kaplan-Meier method, as illustrated in Figure 3. Sepsis patients in HL group experienced a higher incidence of MODS and mortality. The Log-Rank test indicated remarkable differences in the occurrence of MODS and mortality between sepsis patients in HL and LL groups (*P*<0.05).

**Figure 4:**
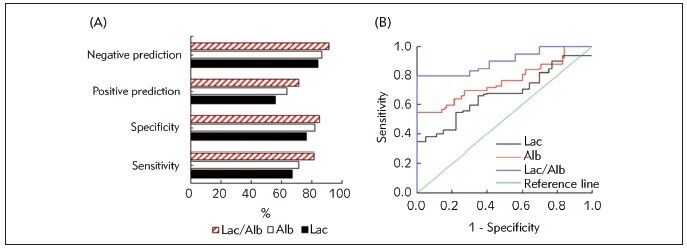
Predictive value of Lac/Alb for prognosis of sepsis patients (A: predictive values; B: ROC curves).

Figure 4 illustrated the evaluation of MODS prediction in sepsis patients based on Lac, Alb, and Lac/Alb levels 48 hours after admission. When employing Lac with a cutoff value 6.82 mmol/L for predicting MODS development in sepsis patients, it demonstrated a sensitivity, specificity, PPV, and NPV of 67.35%, 76.58%, 55.93%, and 84.16%, respectively. By taking Alb (17.58 g/L) to predict the MODS development, values of above four indicators were 71.43%, 81.98%, 63.64%, and 86.67%, respectively. In contrast, when utilizing Lac/Alb (0.45) as an index to predict the MODS development, it showed a sensitivity, specificity, PPV, and NPV of 81.63%, 85.59%, 71.43%, and 91.35%, respectively. Furthermore, the AUC for predicting MODS development in sepsis patients based on Lac, Alb, and Lac/Alb levels were 0.71, 0.80, and 0.89, respectively. Consequently, Lac/Alb experienced a higher predictive efficiency for MODS development in sepsis compared to Lac and Alb.

**Figure 5:**
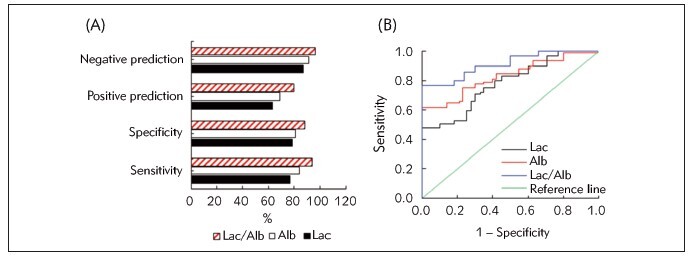
Predictive value of Lac/Alb for mortality of sepsis patients (A: predictive values; B: ROC curves).

Figure 5 depicted the assessment of predicting mortality of sepsis patient using Lac, Alb, and Lac/Alb levels 48 hours after admission. When employing Lac (6.82 mmol/L) for predicting the mortality, it demonstrated a sensitivity, specificity, PPV, and NPV of 76.92%, 78.70%, 63.49%, and 87.63%, respectively. Taking Alb (17.58 g/L) as an index for mortality prediction, the corresponding values of above four indicators were 84.62%, 81.48%, 68.75%, and 91.67%, respectively. When utilizing Lac/Alb (0.45) for mortality prediction, the sensitivity, specificity, PPV, and NPV were 94.23%, 88.89%, 80.33%, and 96.97%, respectively. Moreover, the AUC for predicting mortality of sepsis patient based on Lac, Alb, and Lac/Alb levels were 0.78, 0.85, and 0.91, respectively. These outcomes suggested that Lac/Alb exhibited a higher predictive efficiency for adverse outcomes in sepsis patients than Lac and Alb.

### Correlations of Lac/Alb to APACHE II and SOFA scores of sepsis patients

**Figure 6:**
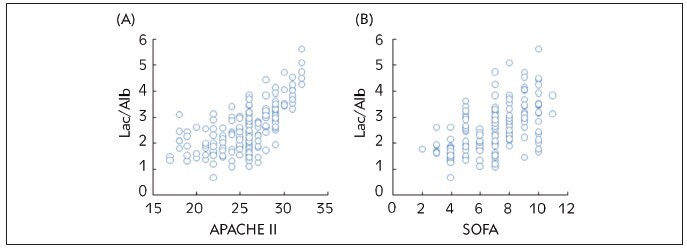
Correlations of Lac/Alb to APACHE II and SOFA scores of sepsis patients.

Figure 6 illustrated the extremely positive correlations between Lac/Alb and both the APACHE II score (r=0.718, *P*<0.05) (Figure 6A) and the SOFA score (r=0.808, *P*<0.05) (Figure 6B) in sepsis patients.

## Discussion

Sepsis is caused by infection, and it can be classified into different severity levels, including uncomplicated sepsis, severe sepsis, and septic shock. Previous research has demonstrated that sepsis is primarily characterized by an inflammatory response that spans the entire pathological process [14] [15] [16]. The release of inflammatory mediators and infiltration of inflammatory cells lead to a cascade release of inflammatory cytokines, ultimately causing a systemic inflammatory response [17]. This response results in severe damage to cellular structures and functions and can lead to dysfunction of local tissues or organs. Additionally, worsening inflammation can result in tissue ischemia, hypoxia, inadequate perfusion, and coagulation abnormalities [18] [19] [20]. If sepsis patients do not receive timely and effective treatment, the condition may progress to MODS or even death. Today, sepsis remains a significant factor contributing to mortality among critically ill patients. The incidence of MODS and mortality associated with sepsis has been increasing over the years, with mortality rates as high as 40% to 80% [21] [22], so that it is the factor with the highest influence on death in ICU [23]. Therefore, identifying effective markers for sepsis diagnosis is of paramount importance for treatment planning and prognosis prediction.

Lac is a product of the body’s glucose metabolism and can reflect tissue perfusion status and the degree of cellular hypoxia. During the onset of sepsis, patients often experience tissue hypoxia, which leads to disruptions in cellular metabolism and microcirculation in tissues [24]. Ischemia and hypoxia can induce the tricarboxylic acid cycle, leading to an upregulation in Lac levels. Additionally, immunoglobulin deficiencies and a downregulation in Alb levels can occur, increasing the risk of mortality in patients [25] [26]. Albumin plays a role in antioxidation, anti-inflammation, and maintaining the integrity of vascular endothelial function [27] [28] [29] [30]. This can help alleviate complications caused by infections and reduce the risk of organ dysfunction. This work disclosed that both MODS patients and deceased patients with sepsis had significantly elevated Lac levels and Lac/Alb ratios, while Alb levels were significantly decreased. As sepsis progresses and becomes more severe, the infection cannot be effectively controlled, leading to multiple organ failure or shock [31]. Various factors, such as increased vascular leakage and decreased synthesis capacity, contribute to decreased Alb levels in sepsis patients, resulting in hypoalbuminemia [32] [33]. This work confirmed that sepsis patients with adverse outcomes had higher Lac/Alb levels. This may be attributed to the fact that in deteriorating sepsis patients, the monocyte-macrophage system and neutrophils generate a large number of inflammatory factors. Increased vascular permeability can lead to capillary leakage, causing an increase in Lac levels [34] [35]. Simultaneously, Alb leakage into the tissue interstitium can cause hypoalbuminemia, further worsening the patient's condition and leading to a poor prognosis.

This work found that in comparison to sepsis patients in LL group, those in HL group possessed higher APACHE II scores, SOFA scores, MVR, D-D levels, and Cre levels, while having lower CVP, PaO_2_/FiO_2_, ScvO_2_, and eGFR levels. Higher APACHE II scores are associated with lower PaO_2_/FiO_2_ levels in sepsis patients. Results suggest that there is a certain correlation between Lac/Alb ratios and the progression of sepsis in patients. Subsequently, this work further analyzed the predictive value of Lac, Alb, and Lac/Alb ratios for the development of MODS or mortality in sepsis patients using ROC curves. The findings indicated that Lac/Alb had higher sensitivity, specificity, PPV, NPV, and AUC when they were employed for prediction of the MODS development or mortality in patients compared to Lac and Alb alone. Lac concentration reflects the interaction between lactate generation and clearance. Sepsis patients with concurrent liver failure may exhibit hyperlactatemia or decreased albumin levels. Additionally, in skeletal muscle, adrenaline stimulates Na^+^/K^+^ adenosine activity and ketone acid metabolism, leading to an increase in plasma lactate levels. Therefore, using lactate alone may not accurately predict the prognosis of sepsis patients [36]. These findings suggest that an elevated Lac/Alb ratio may serve as an early warning signal for the development of MODS or mortality in sepsis patients, providing valuable insights for clinical treatment. Previous research has confirmed the association between APACHE II scores, PaO_2_/FiO_2_, and the development of MODS in sepsis patients. The APACHE II score is a scoring system that takes into account patient physiological indicators, age, and chronic health conditions to assess prognosis and disease severity. Higher scores are indicative of more severe disease and potentially poorer outcomes. SOFA is commonly utilized to monitor organ function in patients suffering from sepsis or septic shock. It can assess disease severity and prognosis. Furthermore, this work analyzed the correlation between Lac/Alb ratios and APACHE II scores as well as SOFA scores, and found that Lac/Alb had a significant positive link with both APACHE II and SOFA scores. This further underscores the close relationship between elevated Lac/Alb ratios and progression of MODS and mortality in sepsis patients, making it an effective marker for predicting prognosis in sepsis patients.

However, this work was subjected to some limitations. Since critically ill sepsis patients received treatment early in the emergency department, the standardization of early goal-directed therapy may also affect Lac/Alb levels, the incidence of MODS, and mortality. Additionally, this work was a single-center case-control study, and further large-scale, multi-center prospective studies are essential for a validation of results in this work.

## Conclusion

In summary, monitoring changes in Lac, Alb, and Lac/Alb levels has a certain value in assessing the severity and prognosis of sepsis patients. Elevated Lac levels, a high Lac/Alb ratio, and low Alb levels indicate poor prognosis in sepsis patients, with Lac/Alb being more efficient in predicting the development of MODS and mortality.

## Dodatak

### Conflict of interest statement

All the authors declare that they have no conflict of interest in this work.
